# Further investigation on the nitration of BODIPY with cupric nitrate: crystal structures of 4,4-di­fluoro-1,3,5,7,8-penta­methyl-2-nitro-4-bora-3a,4a-di­aza-*s*-indacene, 4,4-di­fluoro-3-nitro-8-phenyl-4-bora-3a,4a-di­aza-*s*-indacene, and 3-chloro-6-ethyl-5,7,8-trimethyl-2-nitro-4,4-diphenyl-4-bora-3a,4a-di­aza-*s*-indacene

**DOI:** 10.1107/S2056989017018564

**Published:** 2018-01-09

**Authors:** Dhruval J. Joshi, Meesook Jun, Lijing Yang, Alan J. Lough, Hongbin Yan

**Affiliations:** a Department of Chemistry, Brock University, 1812 Sir Isaac Brock Way, St., Catharines, ON, L2S 3A1, Canada; b Department of Chemistry, University of Toronto, Toronto, Ontario, M5S 3H6, Canada

**Keywords:** BODIPY, nitration, cupric nitrate, regioselectivity, X-ray crystal structure

## Abstract

The treatment of non-fully substituted 4,4-di­fluoro-4-bora-3a,4a-di­aza-*s*-indacene (BODIPY) with cupric nitrate leads to the introduction of a nitro group at different positions of the BODIPY core, depending on the substitution pattern. This methodology complements the treatment of fully substituted BODIPY with cupric nitrate that was previously reported. The crystal structures of 4,4-di­fluoro-1,3,5,7,8-penta­methyl-2-nitro-4-bora-3a,4a-di­aza-*s*-indacene, C_14_H_16_BF_2_N_3_O_2_ (**5a**) 4,4-di­fluoro-3-nitro-8-phenyl-4-bora-3a,4a-di­aza-*s*-indacene, C_15_H_10_BF_2_N_3_O_2_ **(5b**) and 3-chloro-6-ethyl-5,7,8-trimethyl-2-nitro-4,4-diphenyl-4-bora-3a,4a-di­aza-*s*-indacene, C_26_H_25_BClN_3_O_2_ (**5d**) are presented. In all three structures, the fused ring system is in a very flattened ‘V-shape’, with dihedral angles between the two outer five membered rings of 8.12 (14), 6.67 (9) and 12.30 (18) Å for **5a**, **5b** and **5d**, respectively. In each case, the central six-membered ring is in a flattened sofa conformation. In the crystal of **5a**, mol­ecules are linked by weak C—H⋯O and C—H⋯F hydrogen bonds forming sheets parallel to (10-1). In the crystal of **5b** mol­ecules are linked by weak C—H⋯O and C—H⋯F hydrogen bonds and π–π inter­actions forming sheets parallel to (001). In the crystal of **5d**, weak C—H⋯O hydrogen bonds link mol­ecules into chains along [001]. In compound **5d**, the atoms of the nitro group were refined as disordered over two sets of sites with occupancies 0.618 (12) and 0.382 (12).

## Abstract 2

The treatment of non-fully substituted 4,4-di­fluoro-4-bora-3a,4a-di­aza-*s*-indacene (BODIPY) with cupric nitrate leads to the introduction of a nitro group at different positions of the BODIPY core, depending on the substitution pattern. This methodology complements the treatment of fully substituted BODIPY with cupric nitrate that was previously reported. In all three structures, the fused ring system is in a very flattened ‘V-shape’ and the central six-membered ring adopts a flattened sofa conformation.

## Chemical context   

In recent years, 4,4-di­fluoro-4-bora-3a,4a-di­aza-*s*-indacene (BODIPY) has been recognized as an attractive fluoro­phore due to its unique photochemical properties (Ulrich *et al.*, 2008 ▸; Loudet & Burgess, 2007 ▸; Ziessel *et al.*, 2007 ▸). Applications of BODIPY in labeling biomolecules such as peptides and proteins, nucleic acids, and lipids, as well as in material sciences have been explored quite extensively (Ulrich *et al.*, 2008 ▸; Loudet & Burgess, 2007 ▸; Ziessel *et al.*, 2007 ▸; Tram *et al.*, 2011 ▸; Lu *et al.*, 2014 ▸; Bessette & Hanan, 2014 ▸). In order to broaden its utilities, the discovery of reactions to introduce functional group into BODIPY has attracted significant inter­est. Among these, installation of nitro groups into BODIPY core represents a useful approach to functionalize BODIPY (Ulrich *et al.*, 2012 ▸; Esnal *et al.*, 2013 ▸; Gupta *et al.*, 2013 ▸). In this respect, while BODIPY fluoro­phores with nitro groups are poorly fluorescent, their fluorescence is usually restored upon reduction of nitro to amine (Yang *et al.*, 2014 ▸; Yang *et al.*, 2017 ▸). We previously reported the treatment of fully substituted BODIPY, 4,4-di­fluoro-1,3,5,7,8-penta­methyl-2,6-diethyl-4-bora-3a,4a-di­aza-*s*-indacene **1** with cupric nitrate under various conditions (Yang *et al.*, 2014 ▸), leading to the introduction of nitro-, nitro­methyl-, hy­droxy­methyl- and carb­oxy­aldehyde into BODIPY (see Scheme below).
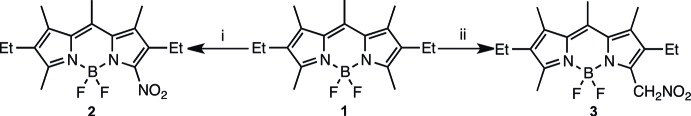


### Reactions between non-fully substituted BODIPY and cupric nitrate   

We report herein that treatment of BODIPY, where at least one of the *R*_1_–*R*_7_ is H, with cupric nitrate leads to the nitration of the BODIPY core (see Scheme below).
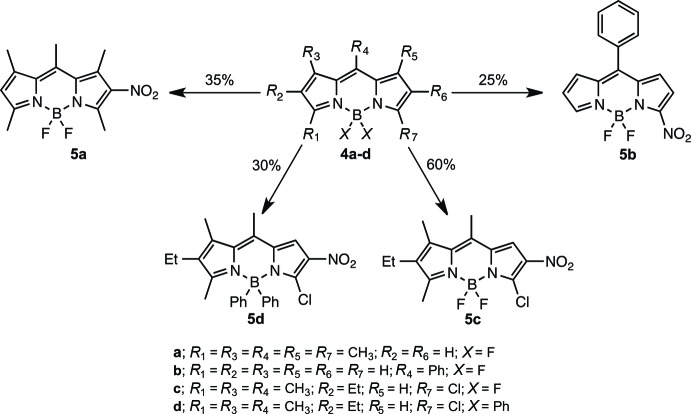


Thus, treatment of 4,4-di­fluoro-1,3,5,7,8-penta­methyl-4-bora-3a,4a-di­aza-*s*-indacene **4a** with cupric nitrate led to the formation of 4,4-di­fluoro-1,3,5,7,8-penta­methyl-2-nitro-4-bora-3a,4a-di­aza-*s*-indacene **5a** as the main product. Similar pattern of nitration was seen in the case of **4c–d**. Reaction of 4,4-di­fluoro-8-phenyl-4-bora-3a,4a-di­aza-*s*-indacene **4b** with cupric nitrate, however, led to the isolation of 4,4-di­fluoro-8-phenyl-3-nitro-4-bora-3a,4a-di­aza-*s*-indacene **5b** as the main product.
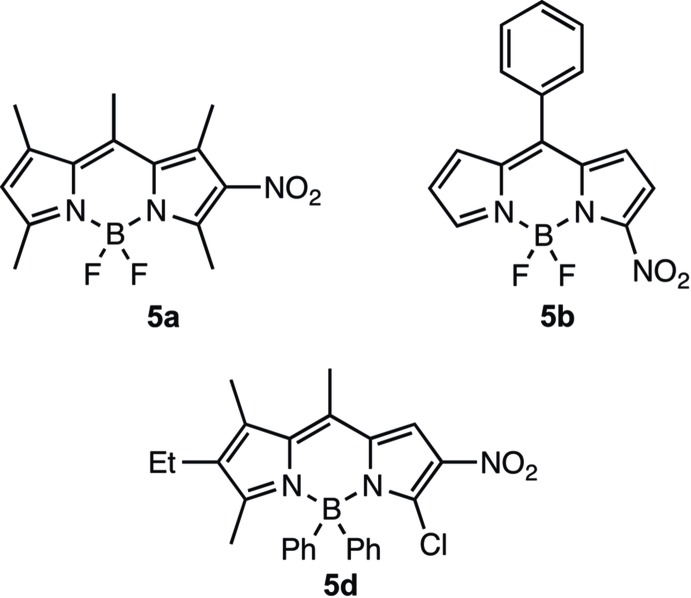


## Structural commentary   

**Figure 1:**
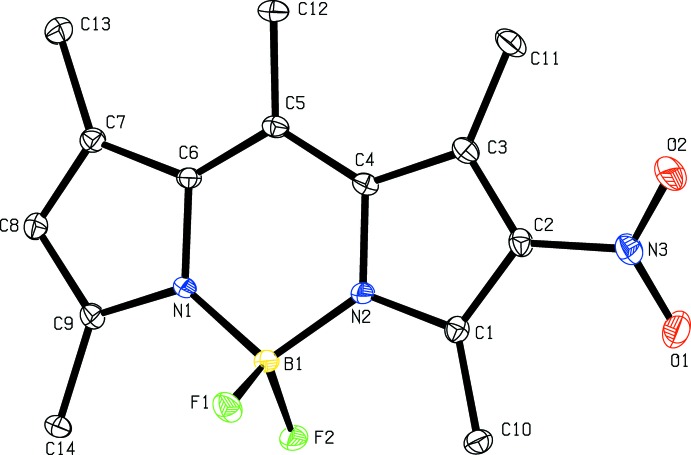
The mol­ecular structure of **5a** with displacement ellipsoids drawn at the 30% probability level. H atoms are not shown.

The mol­ecular structures of **5a**, **5b** and **5d** are shown in Figs. 1 ▸, 2 ▸ and 3 ▸, respectively. In all three structures the fused ring system is in a very flattened ‘V-shape’ with the two outer five-membered rings (N1/C6–C9 and N2/C1–C4) forming dihedral angles of 8.12 (14), 6.67 (9) and 12.30 (18) Å for **5a**, **5b** and **5d**, respectively. The central six-membered ring in each compound forms a flattened sofa conformation with five of the ring atoms (N1/N2/C4/C5/C6), forming an approximate plane with atom B1 displaced from this plane by 0.183 (2), 0.115 (2) and 0.341 (1) Å in **5a**, **5b** and **5d**, respectively. In compound **5d** the nitro group is disordered over two sets of sites with refined occupancies of 0.618 (12) and 0.382 (12). In **5a** the mean plane of the nitro group N3/O1/O2 forms a dihedral angle of 23.9 (2)° with the plane of the N2/C1–C4 ring. The corres­ponding dihedral angles in **5b** and **5d** are 8.47 (17) and 39.8 (8)° [with a value of 18.2 (14)° for the minor component of disorder]. In **5d** the dihedral angle between the two phenyl rings (C15–C20 and C21–C26) is 53.72 (7)°. In **5b** the phenyl ring (C10–C15) forms a dihedral angle of 53.94 (7)° with the five essentially planar atoms (N1/N2/C4/C4/C6) of the central six-membered ring. The orientation of the phenyl rings in **5b** and **5d** presumably alleviates any steric inter­action between H atoms of the fused ring system and the phenyl ring(s).

## Supra­molecular features   

**Figure 4:**
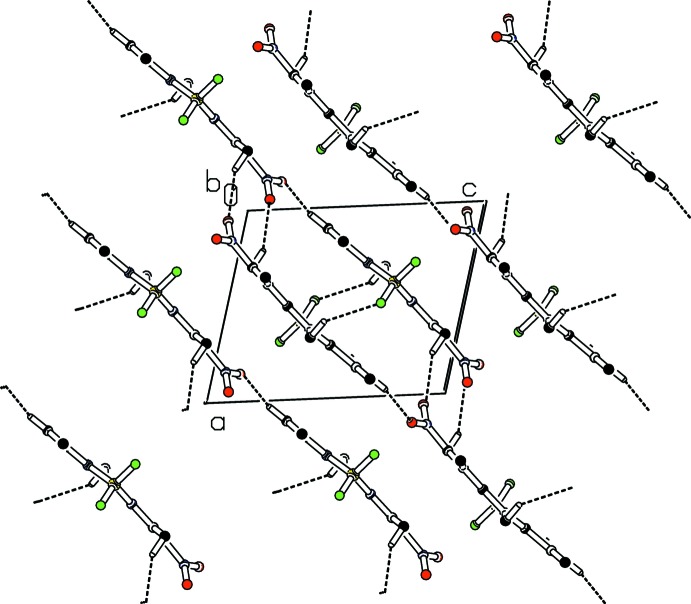
Part of the crystal structure of **5a** with weak C—H⋯O and C—H⋯F hydrogen bonds shown as dashed lines. Only H atoms involved in hydrogen bonds are shown.

**Figure 5:**
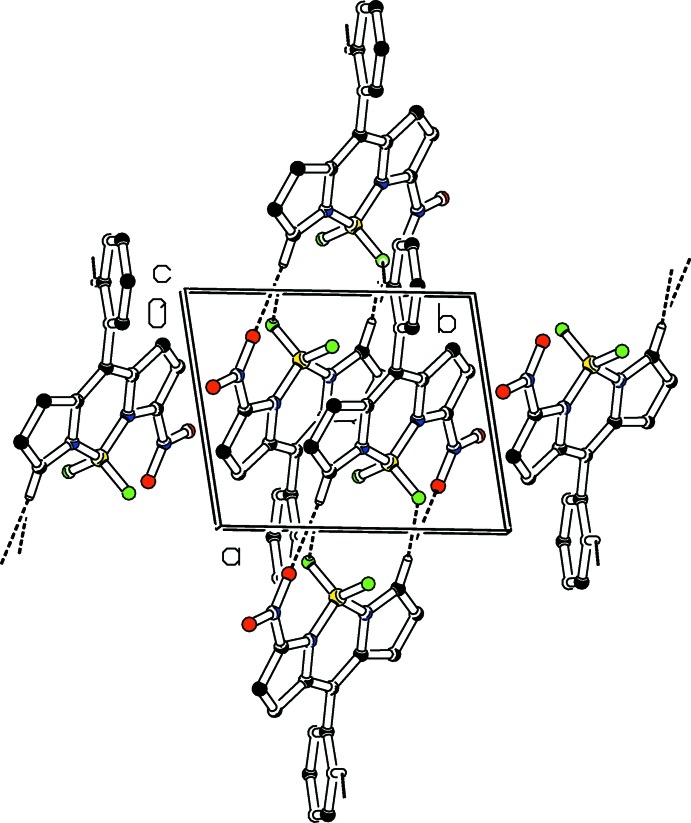
Part of the crystal structure of **5b** with weak C—H⋯O and C—H⋯F hydrogen bonds shown as dashed lines. Only H atoms involved in hydrogen bonds are shown.

**Figure 6:**
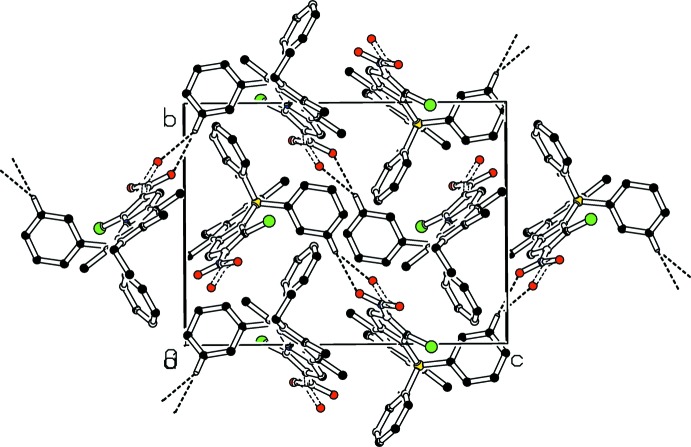
Part of the crystal structure of **5d** with weak C—H⋯O hydrogen bonds shown as dashed lines. Only H atoms involved in hydrogen bonds are shown. Both components of disorder are shown.

In the crystal of **5a**, weak C—H⋯O and C—H⋯F hydrogen bonds link the mol­ecules forming ‘double’ sheets (Table 1 ▸, Fig. 4 ▸) parallel to (10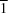) and within these sheets there are π–π stacking inter­actions with a centroid–centroid distance of *Cg*1⋯*Cg*1(−*x* + 1, −y + 1, −*z* + 1) = 3.870 (1) Å, where *Cg*1 is the centroid of all atoms in the fused ring system (B1/N1/N2/C1–C9). In the crystal of **5b**, weak bifurcated C—H⋯(O,F) and C—H⋯F hydrogen bonds link the mol­ecules forming chains (Table 2 ▸, Fig. 5 ▸) along [100]. In addition π–π inter­actions with a centroid–centroid distance of *Cg*2⋯*Cg*2(−*x* + 1, −*y* + 2, −*z* + 1) = 3.435 (1) Å connect the chains into sheets parallel to (001), where *Cg*2 is the centroid of the ring atoms N2/C1–C4. In the crystal of **5d**, weak C—H⋯O hydrogen bonds link mol­ecules forming zigzag chains along [001] (Table 3 ▸, Fig. 6 ▸). There are no significant π–π inter­actions in compound **5d**.

## Database survey   

A survey of the Cambridge Structural Database (V5.38, last update May 2017; Groom *et al.*, 2016 ▸) revealed that the crystal structure of 4,4-di­fluoro-1,3,5,7,8-penta­methyl-4-bora-3a,4a-di­aza-*s*-indacene has been determined at three different temperatures *viz.* JEHFUX at 295 K (Picou *et al.*, 1990 ▸) JEHFUX01 at 200 K (Choi *et al.* 2014 ▸) and JEHFUX02 at 100 K (Wang *et al.*, 2014 ▸). This structure corresponds to compound **5a** without the nitro substituent and in all three equivalent literature structures, the atoms of the fused-ring system lie on a crystallographic mirror plane and hence the fused-ring system is exactly planar. In the compound corres­ponding to **5b** without the nitro substituent, *viz*. 4,4-di­fluoro-8-phenyl-4-bora-3a,4a-di­aza-*s*-indacene (VAWDED, Kee *et al.*, 2005 ▸), the mol­ecule is bis­ected by a crystallographic twofold rotation axis through the central B and C atoms of the six-membered ring and the six-membered ring is essentially planar. To date, compound **5d** is the only crystal structure with a 4-bora-3a,4a-di­aza-*s*-indacene core which is substituted by two phenyl rings at boron and a Cl atom in the 3-position.

## Synthesis and crystallization   

^1^H, ^13^C, ^11^B, and ^19^F NMR spectra were recorded at 400.2, 100.6, 128.4, and 376.6 MHz, respectively, with a Bruker AV400 spectrometer; *J* values are given in Hz. Chemical shifts are given in ppm. High-resolution mass spectra were measured with a ThermoFisher high resolution Double Focusing magnetic sector mass spectrometer.

Chemicals were purchased from Aldrich or TCI America and used without further purification unless stated otherwise. Tri­ethyl­amine was dried by heating under reflux in the presence of calcium hydride and distilled in an atmosphere of nitro­gen. Silica gel (SiliCycle, >230 mesh) was used for flash chromatography. Thin layer chromatography was performed on SiliCycle SiliaPlate F-254 TLC plates, with the following system: ethyl­acetate–hexane (3:7 *v*/*v*).

### Synthesis of BODIPY starting materials   

**3-Chloro-4,4-di­fluoro-6-ethyl-5,7,8-trimethyl-4-bora-3a,4a-di­aza-** ***s*** **-indacene 4c**

To a solution of 2-acetyl-5-chloro­pyrrole (Leen *et al.*, 2011 ▸) (325 mg, 2.27 mmol) in di­chloro­methane (10 mL) under nitro­gen was added 3-ethyl-2,4-di­methyl­pyrrole (310 µL, 2.30 mmol) and the resulting solution was cooled (ice–water bath), followed by the addition of POCl_3_ (220 µL, 2.36 mmol). After the reaction mixture was stirred at room temperature for 6 h, tri­ethyl­amine (3.2 mL, 23 mmol) was added and the mixture was stirred for 10 min. Upon cooling (ice–water bath), boron trifluoride diethyl etherate (3.1 mL, 25 mmol) was added dropwise and the reaction mixture was stirred at room temperature for 1 h. The orange solution was diluted with diethyl ether (200 mL) and extracted with water (3 × 100 mL). The organic layer was dried (MgSO_4_) and concentrated under reduced pressure. The residue was then purified by column chromatography on silica gel. The appropriate fractions, which were eluted with di­chloro­methane–hexane (70:30 *v*/*v*), were combined and evaporated under reduced pressure to give the title compound as an orange solid (500 mg, 74%). *R*_f_: 0.52. δ_H_(CDCl_3_): 1.08 (3 H, *t*, *J* = 7.5), 2.35 (3 H, *s*), 2.44 (2 H, *q*, *J* = 7.5), 2.52 (3 H, *s*), 2.60 (3 H, *s*), 6.28 (1 H, *d*, *J* = 3.9), 6.98 (1 H, *s*, *J* = 3.9); δ_C_(CDCl_3_): 13.1, 14.0, 14.5, 15.8, 17.1, 114.5, 122.8, 132.5, 133.1, 134.0, 135.7, 138.6, 140.7, 161.1. δ_B_(CDCl_3_): 0.41 (*t*, *J* = 31); δ_F_(CDCl_3_): −147.2 (*q*, *J* = 31). C_14_H_16_BClF_2_N_2_ requires 296.10631, found (EI) 296.1059.

**3-Chloro-4,4-diphenyl-6-ethyl-5,7,8-trimethyl-4-bora-3a,4a-di­aza-** ***s*** **-indacene 4d**

To a solution of 2-acetyl-5-chloro­pyrrole (400 mg, 2.80 mmol) in di­chloro­methane (8 mL) under an atmosphere of nitro­gen was added 2,4-di­methyl­pyrrole (380 µL, 3.69 mmol) and the resulting solution was cooled (ice–water bath), followed by addition of POCl_3_ (260 µL, 2.80 mmol). After the solution was stirred at room temperature for 6 h, tri­ethyl­amine (1.0 mL, 7.2 mmol) was added and the mixture was stirred for 10 min. Diphenyl boronbromide (Nöth & Vahrenkamp, 1968 ▸) (1.35 g, 5.53 mmol) was then added dropwise while the reaction mixture was cooled (ice–water bath). After the reaction mixture had been stirred at room temperature for 1 h, the orange products were poured into diethyl ether (200 mL) and extracted with water (3 × 100 mL). The organic layer was dried (MgSO_4_) and concentrated under reduced pressure. The product was purified by flash column chromatography on silica gel. The appropriate fractions, which were eluted with di­chloro­methane–hexane (30:70 *v*/*v*), were combined and evaporated under reduced pressure to give the title compound as an orange solid (780 mg, 68%). *R*_f_: 0.66. δ_H_(CDCl_3_): 1.02 (3 H, *t*, *J* = 7.5), 1.78 (3 H, *s*), 3.92 (2 H, *q*, *J* = 7.5), 2.44 (3 H, *s*) 2.64 (3 H, *s*), 6.22 (1 H, *d*, *J* = 4.2), 7.05 (1 H, *s*, *J* = 4.2), 7.18–7.39 (10 H, *m*). δ_C_(CDCl_3_): 14.4, 14.7, 15.2, 16.5, 17.4, 114.9, 121.1, 125.8, 127.1, 133.0, 133.9, 135.5, 136.3, 137.4, 138.8, 159.1. δ_B_(CDCl_3_): 0.33. C_26_H_26_BClN_2_ requires 412.18776, found (EI) 412.1867.

### General procedure for the treatment of 4a–e with cupric nitrate   

To a solution of BODIPY (100 mg) in anhydrous CH_2_Cl_2_ (20 mL), a solution of Cu(NO_3_)_2_·3H_2_O (5 mol. equiv.) in anhydrous MeCN (10 mL) was added. The reaction mixture was stirred at room temperature and the reaction progress was monitored by TLC. Upon complete consumption of starting materials, the products were evaporated under reduced pressure. The residue was redissolved in CH_2_Cl_2_ (20 mL) and extracted with water (320 mL). The organic layer was collected, dried (MgSO_4_), and evaporated under reduced pressure. The residue was purified by column chromatography on silica gel. The appropriate fractions, eluted with CH_2_Cl_2_–hexane, were combined and evaporated under reduced pressure to give the nitro BODIPY.

### Synthesis of 5a–d   

**4,4-Di­fluoro-1,3,5,7,8-penta­methyl-2-nitro-4-bora-3a,4a-di­aza-** ***s*** **-indacene 5a**

Treatment of 4,4-di­fluoro-1,3,5,7,8-penta­amethyl-4-bora-3a,4a-di­aza-*s*-indacene **4a** (Bandichhor *et al.*, 2006 ▸) with cupric nitrate under the conditions described in the general procedure for 10 min led to the isolation of 4,4-di­fluoro-1,3,5,7,8-penta­methyl-2-nitro-4-bora-2-nitro-3a,4a-di­aza-*s*-indacene **5a** as the main product (35% yield). *R*_f_: 0.30. δ_H_(CDCl_3_): 2.51 (3 H, *s*), 2.62 (3 H, *s*), 2.72 and 2.73 (6 H, two *s*), 2.83 (3 H, *s*), 6.32 (1 H, *s*). δ_C_(CDCl_3_): 14.1, 14.4, 15.1, 17.7, 18.0, 125.2, 128.2, 132.0, 135.9, 138.9, 143.7, 146.8, 147.7, 162.5. δ_F_(CDCl_3_): −144.5 (*q*, *J* = 31.6). δ_B_(CDCl_3_): 0.38 (*t*, *J* = 31.6). C_14_H_16_BF_2_N_3_O_2_ requires 307.13036, found (EI): 307.1298. Orange needles of **5a** were recrystallized from mixed solvents of hexa­nes/chloro­form.

**4,4-Di­fluoro-8-phenyl-3-nitro-4-bora-3a,4a-di­aza-** ***s*** **-indacene 5b**

Treatment of 4,4-di­fluoro-8-phenyl-4-bora-3a,4a-di­aza-*s*-indacene **4b** (Rao *et al.*, 2011 ▸) with cupric nitrate under the conditions described in the general procedure for 60 min led to the isolation of 4,4-di­fluoro-2-nitro-8-phenyl-4-bora-2-nitro-3a,4a-di­aza-*s*-indacene **5a** as the main product (25%). *R*_f_: 0.24. δ_H_(CDCl_3_): 6.79 (1 H, *d*, *J* = 4.1), 6.84 (1 H, *d*, *J* = 4.1), 7.21 (2 H, *t*, *J* = 4.4), 7.56–7.71 (5 H, *m*), 8.36 (1 H, *s*). δ_C_(CDCl_3_): 114.9, 123.8, 126.6, 128.9, 130.6, 131.7, 132.6, 134.3, 136.2, 137.9, 149.1, 150.7, 153.6. δ_B_(CDCl_3_): 0.36 (*t*, *J* = 25). δ_F_(CDCl_3_): −144.0 (*q*, *J* = 25). C_15_H_10_BF_2_N_3_O_2_ requires 313.08341, found (EI) 313.0832. Orange plates of **5b** were recrystallized from mixed solvents of hexa­nes/chloro­form.

**3-Chloro-4,4-di­fluoro-6-ethyl-5,7,8-trimethyl-2-nitro-4-bora-3a,4a-di­aza-** ***s*** **-indacene 5c**

Treatment of 3-chloro-4,4-di­fluoro-6-ethyl-5,7,8-trimethyl-4-bora-3a,4a-di­aza-*s*-indacene **4c** with cupric nitrate under the conditions described in the general procedure for 1 d led to the isolation of **5c** as the main product (60%). *R*_f_: 0.24. δ_H_(CDCl_3_): 1.13 (3 H, *t*, *J* = 7.6), 2.42 (3 H, *s*), 2.49 (2 H, *q*, *J* = 7.6), 2.59 (3 H, *s*), 2.68 (3 H, *s*), 7.50 (1 H, *s*). δ_C_(CDCl_3_): 13.8, 14.1, 14.4, 15.5, 17.1, 115.2, 129.4, 130.2, 137.3, 137.5, 139.11, 139.13, 143.3, 168.7. δ_F_(CDCl_3_): −146.3 (*t*, *J* = 29.6). δ_B_(CDCl_3_): 0.19 (*t*, *J* = 29.6). C_14_H_15_BClF_2_N_3_O_2_ requires 341.09139, found (EI): 341.0907.

**3-Chloro-4,4-diphenyl-6-ethyl-5,7,8-trimethyl-2-nitro-4-bora-3a,4a-di­aza-** ***s*** **-indacene 5d**

Treatment of 3-chloro-4,4-diphenyl-6-ethyl-5,7,8-trimethyl-4-bora-3a,4a-di­aza-*s*-indacene **4d** with cupric nitrate under the conditions described in the general procedure for 4 h led to the isolation of **5d** as the main product (30%). *R*_f_: 0.46. δ_H_(CDCl_3_): 1.03 (3 H, *t*, *J* = 7.6), 1.87 (3 H, *s*), 2.41 (2 H, *q*, *J* = 7.6), 2.48 (3 H, *s*), 2.70 (3 H, *s*), 7.23–7.28 (6 H, *m*), 7.36–7.39 (4 H, *m*), 7.61 (1 H, *s*). δ_C_(CDCl_3_): 14.2, 14.7, 15.8, 16.1, 17.4, 114.5, 126.5, 127.5, 129.5, 130.6, 133.7, 135.1, 137.6, 137.8, 139.3, 140.2, 166.7. δ_B_(CDCl_3_): 1.08 (*br*). C_26_H_25_BClN_3_O_2_ requires 457.17284, found (EI) 457.1733. Orange blocks of **5d** were recrystallized from mixed solvents of hexa­nes/chloro­form.

## Refinement   

Crystal data, data collection and structure refinement details are summarized in Table 4 ▸. In all three compounds, the H atoms were placed in calculated positions and included in the refinement in a riding-model approximation with *U*_iso_(H) = 1.2*U*_eq_(C) or 1.5*U*_eq_(C_meth­yl_). In compound **5d** the atoms of the nitro group were refined as disordered over two sets of sites with occupancies 0.618 (12) and 0.382 (12).

In the refinement, restraints were applied to the bond distances of the nitro group so that those in the minor component of disorder were similar to those in the major component. The refinement of the minor component of disorder was also restrained to be approximately planar. These restraints were achieved using the SADI and FLAT commands in *SHELXL* (Sheldrick, 2015*b* ▸).

## Supplementary Material

Crystal structure: contains datablock(s) 5a, 5b, 5d. DOI: 10.1107/S2056989017018564/hb7723sup1.cif

Structure factors: contains datablock(s) 5a. DOI: 10.1107/S2056989017018564/hb77235asup2.hkl

Structure factors: contains datablock(s) 5b. DOI: 10.1107/S2056989017018564/hb77235bsup3.hkl

Structure factors: contains datablock(s) 5d. DOI: 10.1107/S2056989017018564/hb77235dsup4.hkl

CCDC references: 1814110, 1814109, 1814108

Additional supporting information:  crystallographic information; 3D view; checkCIF report

## Figures and Tables

**Figure 2:**
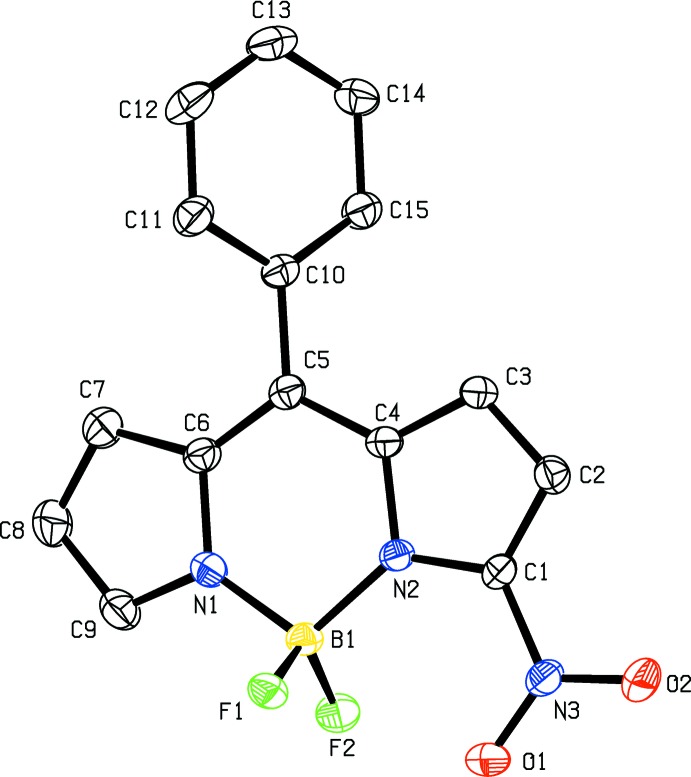
The mol­ecular structure of **5b** with displacement ellipsoids drawn at the 30% probability level. H atoms are not shown.

**Figure 3:**
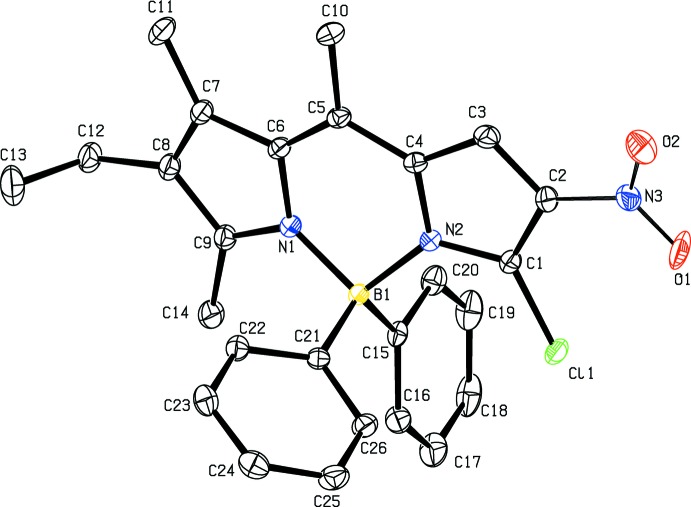
The mol­ecular structure of **5d** with displacement ellipsoids drawn at the 30% probability level. Neither the H atoms not the minor component of disorder are shown.

**Table 1:** Hydrogen-bond geometry (Å, °) for (**5a**)

*D*—H⋯*A*	*D*—H	H⋯*A*	*D*⋯*A*	*D*—H⋯*A*
C8—H8*A*⋯O2^i^	0.95	2.46	3.290 (3)	146
C10—H10*C*⋯O1^ii^	0.98	2.46	3.371 (3)	155
C12—H12*B*⋯F2^iii^	0.98	2.53	3.329 (3)	139

Symmetry codes: (i) 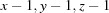; (ii) 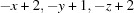; (iii) 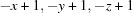.

**Table 2:** Hydrogen-bond geometry (Å, °) for (**5b**)

*D*—H⋯*A*	*D*—H	H⋯*A*	*D*⋯*A*	*D*—H⋯*A*
C9—H9*A*⋯F1^i^	0.95	2.40	3.2788 (18)	155
C9—H9*A*⋯O1^i^	0.95	2.59	3.3420 (19)	136
C15—H15*A*⋯F1^ii^	0.95	2.40	3.2946 (17)	157

Symmetry codes: (i) 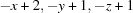; (ii) 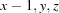.

**Table 3:** Hydrogen-bond geometry (Å, °) for (**5d**)

*D*—H⋯*A*	*D*—H	H⋯*A*	*D*⋯*A*	*D*—H⋯*A*
C19—H19*A*⋯O2^i^	0.95	2.43	3.365 (4)	168
C19—H19*A*⋯O2*A* ^i^	0.95	2.36	3.238 (13)	154

Symmetry code: (i) 
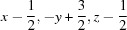
.

**Table 4:** Experimental details

	(**5a**)	(**5b**)	(**5d**)
Crystal data			
Chemical formula	C_14_H_16_BF_2_N_3_O_2_	C_15_H_10_BF_2_N_3_O_2_	C_26_H_25_BClN_3_O_2_
*M*_r_	307.11	313.07	457.75
Crystal system, space group	Triclinic, *P* 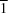	Triclinic, *P* 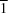	Monoclinic, *P*2_1_/*n*
Temperature (K)	150	150	150
*a*, *b*, *c* (Å)	8.2837 (9), 8.6660 (9), 10.6619 (12)	7.2833 (2), 8.5450 (3), 11.8803 (4)	11.8359 (4), 12.0825 (4), 16.5811 (5)
α, β, γ (°)	110.762 (3), 101.468 (4), 95.463 (3)	81.093 (2), 74.358 (2), 78.581 (2)	90, 104.116 (1), 90
*V* (Å^3^)	689.83 (13)	693.86 (4)	2299.62 (13)
*Z*	2	2	4
Radiation type	Mo *K*α	Cu *K*α	Cu *K*α
μ (mm^−1^)	0.12	1.01	1.70
Crystal size (mm)	0.18 × 0.06 × 0.03	0.12 × 0.08 × 0.03	0.19 × 0.18 × 0.10

Data collection			
Diffractometer	Bruker Kappa APEX-DUO CCD	Bruker Kappa APEX-DUO CCD	Bruker Kappa APEX-DUO CCD
Absorption correction	Multi-scan (*SADABS*, Bruker, 2014 ▸)	Multi-scan (*SADABS*, Bruker, 2014 ▸)	Multi-scan (*SADABS*, Bruker, 2014 ▸)
*T*_min_, *T*_max_	0.681, 0.746	0.661, 0.753	0.586, 0.753
No. of measured, independent and observed [*I* > 2σ(*I*)] reflections	18324, 3194, 2192	21668, 2453, 2109	43708, 4080, 3864
*R*_int_	0.058	0.042	0.047
(sin θ/λ)_max_ (Å^−1^)	0.651	0.598	0.597

Refinement			
*R*[*F* ^2^ > 2σ(*F* ^2^)], *wR*(*F* ^2^), *S*	0.052, 0.144, 1.07	0.033, 0.086, 1.05	0.033, 0.085, 1.04
No. of reflections	3194	2453	4080
No. of parameters	204	208	330
No. of restraints	0	0	8
H-atom treatment	H-atom parameters constrained	H-atom parameters constrained	H-atom parameters constrained
Δρ_max_, Δρ_min_ (e Å^−3^)	0.39, −0.24	0.14, −0.24	0.27, −0.29

Computer programs: *APEX2* (Bruker, 2014 ▸), *APEX2*, *SAINT* (Bruker, 2014 ▸), *SHELXT* (Sheldrick, 2015*a* ▸), *SHELXL2014*/7 (Sheldrick, 2015*b* ▸), *SHELXL2016/6* (Sheldrick, 2015*b* ▸), *PLATON* (Spek, 2009 ▸), *SHELXTL* (Sheldrick, 2008 ▸).

## supplementary crystallographic information

### 4,4-Difluoro-1,3,5,7,8-pentamethyl-2-nitro-4-bora-3a,4a-diaza-s-indacene (5a) Crystal data

**Table:**

C_14_H_16_BF_2_N_3_O_2_	*Z* = 2
*M**_r_* = 307.11	*F*(000) = 320
Triclinic, *P*1	*D*_x_ = 1.479 Mg m^−^^3^
*a* = 8.2837 (9) Å	Mo *K*α radiation, λ = 0.71073 Å
*b* = 8.6660 (9) Å	Cell parameters from 4795 reflections
*c* = 10.6619 (12) Å	θ = 2.6–27.6°
α = 110.762 (3)°	µ = 0.12 mm^−^^1^
β = 101.468 (4)°	*T* = 150 K
γ = 95.463 (3)°	Needle, orange
*V* = 689.83 (13) Å^3^	0.18 × 0.06 × 0.03 mm

### 4,4-Difluoro-1,3,5,7,8-pentamethyl-2-nitro-4-bora-3a,4a-diaza-s-indacene (5a) Data collection

**Table:**

Bruker Kappa APEX-DUO CCD diffractometer	2192 reflections with *I* > 2σ(*I*)
Radiation source: sealed tube with Bruker Triumph monochromator	*R*_int_ = 0.058
φ and ω scans	θ_max_ = 27.6°, θ_min_ = 2.1°
Absorption correction: multi-scan (SADABS, Bruker, 2014)	*h* = −10→10
*T*_min_ = 0.681, *T*_max_ = 0.746	*k* = −11→11
18324 measured reflections	*l* = −13→13
3194 independent reflections	

### 4,4-Difluoro-1,3,5,7,8-pentamethyl-2-nitro-4-bora-3a,4a-diaza-s-indacene (5a) Refinement

**Table:**

Refinement on *F*^2^	0 restraints
Least-squares matrix: full	Hydrogen site location: inferred from neighbouring sites
*R*[*F*^2^ > 2σ(*F*^2^)] = 0.052	H-atom parameters constrained
*wR*(*F*^2^) = 0.144	*w* = 1/[σ^2^(*F*_o_^2^) + (0.0736*P*)^2^ + 0.204*P*] where *P* = (*F*_o_^2^ + 2*F*_c_^2^)/3
*S* = 1.07	(Δ/σ)_max_ = 0.001
3194 reflections	Δρ_max_ = 0.39 e Å^−^^3^
204 parameters	Δρ_min_ = −0.24 e Å^−^^3^

### 4,4-Difluoro-1,3,5,7,8-pentamethyl-2-nitro-4-bora-3a,4a-diaza-s-indacene (5a) Special details

**Table:**

Geometry. All esds (except the esd in the dihedral angle between two l.s. planes) are estimated using the full covariance matrix. The cell esds are taken into account individually in the estimation of esds in distances, angles and torsion angles; correlations between esds in cell parameters are only used when they are defined by crystal symmetry. An approximate (isotropic) treatment of cell esds is used for estimating esds involving l.s. planes.

### 4,4-Difluoro-1,3,5,7,8-pentamethyl-2-nitro-4-bora-3a,4a-diaza-s-indacene (5a) Fractional atomic coordinates and isotropic or equivalent isotropic displacement parameters (Å^2^)

**Table:**

	*x*	*y*	*z*	*U*_iso_*/*U*_eq_	
F1	0.30619 (16)	0.28032 (15)	0.74821 (12)	0.0257 (3)	
F2	0.51390 (15)	0.22107 (15)	0.63858 (12)	0.0242 (3)	
O1	0.9463 (2)	0.7217 (2)	1.08010 (18)	0.0364 (5)	
O2	0.8553 (2)	0.9556 (2)	1.11463 (18)	0.0393 (5)	
N1	0.3009 (2)	0.3522 (2)	0.54914 (17)	0.0158 (4)	
N2	0.5130 (2)	0.5113 (2)	0.77068 (17)	0.0161 (4)	
N3	0.8424 (2)	0.8035 (2)	1.05038 (19)	0.0254 (4)	
C1	0.6403 (3)	0.5461 (3)	0.8834 (2)	0.0179 (4)	
C2	0.7004 (3)	0.7192 (3)	0.9354 (2)	0.0195 (5)	
C3	0.6077 (3)	0.7943 (3)	0.8555 (2)	0.0192 (5)	
C4	0.4894 (3)	0.6603 (2)	0.7513 (2)	0.0167 (4)	
C5	0.3633 (3)	0.6568 (2)	0.6372 (2)	0.0169 (4)	
C6	0.2766 (3)	0.5046 (2)	0.5355 (2)	0.0158 (4)	
C7	0.1555 (3)	0.4640 (3)	0.4053 (2)	0.0179 (5)	
C8	0.1121 (3)	0.2929 (3)	0.3481 (2)	0.0196 (5)	
H8A	0.0351	0.2293	0.2615	0.024*	
C9	0.2001 (3)	0.2260 (3)	0.4383 (2)	0.0186 (5)	
C10	0.6895 (3)	0.4176 (3)	0.9389 (2)	0.0224 (5)	
H10A	0.5940	0.3260	0.9097	0.034*	
H10B	0.7241	0.4690	1.0403	0.034*	
H10C	0.7828	0.3732	0.9032	0.034*	
C11	0.6437 (3)	0.9751 (3)	0.8742 (2)	0.0277 (5)	
H11A	0.7646	1.0172	0.9089	0.041*	
H11B	0.5858	1.0410	0.9408	0.041*	
H11C	0.6042	0.9854	0.7850	0.041*	
C12	0.3210 (3)	0.8177 (3)	0.6290 (2)	0.0237 (5)	
H12A	0.2066	0.7970	0.5722	0.036*	
H12B	0.3995	0.8622	0.5871	0.036*	
H12C	0.3295	0.8993	0.7224	0.036*	
C13	0.0903 (3)	0.5777 (3)	0.3373 (2)	0.0238 (5)	
H13A	0.0292	0.5107	0.2411	0.036*	
H13B	0.1843	0.6567	0.3390	0.036*	
H13C	0.0148	0.6400	0.3873	0.036*	
C14	0.1843 (3)	0.0471 (3)	0.4224 (2)	0.0220 (5)	
H14A	0.1568	0.0359	0.5043	0.033*	
H14B	0.2905	0.0092	0.4124	0.033*	
H14C	0.0952	−0.0217	0.3398	0.033*	
B1	0.4081 (3)	0.3348 (3)	0.6781 (2)	0.0175 (5)	

### 4,4-Difluoro-1,3,5,7,8-pentamethyl-2-nitro-4-bora-3a,4a-diaza-s-indacene (5a) Atomic displacement parameters (Å^2^)

**Table:**

	*U*^11^	*U*^22^	*U*^33^	*U*^12^	*U*^13^	*U*^23^
F1	0.0300 (7)	0.0242 (7)	0.0225 (7)	−0.0032 (6)	0.0048 (6)	0.0114 (5)
F2	0.0264 (7)	0.0162 (6)	0.0248 (7)	0.0083 (5)	0.0004 (6)	0.0038 (5)
O1	0.0245 (9)	0.0376 (10)	0.0374 (10)	0.0074 (8)	−0.0032 (8)	0.0086 (8)
O2	0.0478 (12)	0.0204 (9)	0.0319 (10)	−0.0037 (8)	−0.0069 (8)	0.0008 (7)
N1	0.0176 (9)	0.0109 (8)	0.0169 (9)	0.0017 (7)	0.0019 (7)	0.0045 (7)
N2	0.0178 (9)	0.0129 (9)	0.0161 (9)	0.0037 (7)	0.0017 (7)	0.0051 (7)
N3	0.0267 (11)	0.0212 (10)	0.0227 (10)	−0.0004 (8)	0.0034 (8)	0.0044 (8)
C1	0.0180 (10)	0.0189 (11)	0.0149 (10)	0.0037 (8)	0.0036 (8)	0.0046 (8)
C2	0.0192 (11)	0.0182 (11)	0.0167 (10)	0.0017 (9)	0.0028 (9)	0.0026 (8)
C3	0.0218 (11)	0.0156 (11)	0.0187 (10)	0.0019 (9)	0.0069 (9)	0.0043 (8)
C4	0.0203 (11)	0.0128 (10)	0.0170 (10)	0.0028 (8)	0.0058 (8)	0.0053 (8)
C5	0.0182 (10)	0.0152 (10)	0.0205 (11)	0.0039 (8)	0.0077 (9)	0.0089 (9)
C6	0.0173 (10)	0.0135 (10)	0.0182 (10)	0.0038 (8)	0.0045 (8)	0.0077 (8)
C7	0.0177 (11)	0.0192 (11)	0.0192 (10)	0.0032 (8)	0.0055 (9)	0.0097 (9)
C8	0.0179 (11)	0.0206 (11)	0.0184 (10)	0.0018 (9)	0.0013 (9)	0.0075 (9)
C9	0.0194 (11)	0.0160 (11)	0.0177 (10)	0.0016 (8)	0.0035 (9)	0.0044 (8)
C10	0.0267 (12)	0.0198 (11)	0.0199 (11)	0.0068 (9)	0.0015 (9)	0.0083 (9)
C11	0.0345 (13)	0.0153 (11)	0.0280 (12)	−0.0031 (10)	0.0044 (10)	0.0059 (9)
C12	0.0283 (12)	0.0157 (11)	0.0288 (12)	0.0064 (9)	0.0051 (10)	0.0109 (9)
C13	0.0230 (12)	0.0246 (12)	0.0251 (12)	0.0032 (9)	0.0022 (10)	0.0132 (10)
C14	0.0259 (12)	0.0137 (11)	0.0238 (11)	0.0008 (9)	0.0002 (9)	0.0080 (9)
B1	0.0197 (12)	0.0150 (12)	0.0176 (11)	0.0037 (9)	0.0025 (10)	0.0070 (9)

### 4,4-Difluoro-1,3,5,7,8-pentamethyl-2-nitro-4-bora-3a,4a-diaza-s-indacene (5a) Geometric parameters (Å, º)

**Table:**

F1—B1	1.385 (3)	C7—C8	1.367 (3)
F2—B1	1.390 (3)	C7—C13	1.496 (3)
O1—N3	1.229 (2)	C8—C9	1.410 (3)
O2—N3	1.233 (2)	C8—H8A	0.9500
N1—C9	1.345 (3)	C9—C14	1.489 (3)
N1—C6	1.408 (3)	C10—H10A	0.9800
N1—B1	1.546 (3)	C10—H10B	0.9800
N2—C1	1.351 (3)	C10—H10C	0.9800
N2—C4	1.403 (3)	C11—H11A	0.9800
N2—B1	1.550 (3)	C11—H11B	0.9800
N3—C2	1.431 (3)	C11—H11C	0.9800
C1—C2	1.401 (3)	C12—H12A	0.9800
C1—C10	1.488 (3)	C12—H12B	0.9800
C2—C3	1.402 (3)	C12—H12C	0.9800
C3—C4	1.407 (3)	C13—H13A	0.9800
C3—C11	1.500 (3)	C13—H13B	0.9800
C4—C5	1.427 (3)	C13—H13C	0.9800
C5—C6	1.388 (3)	C14—H14A	0.9800
C5—C12	1.497 (3)	C14—H14B	0.9800
C6—C7	1.446 (3)	C14—H14C	0.9800
			
C9—N1—C6	108.30 (17)	C1—C10—H10A	109.5
C9—N1—B1	125.81 (17)	C1—C10—H10B	109.5
C6—N1—B1	125.47 (17)	H10A—C10—H10B	109.5
C1—N2—C4	109.29 (17)	C1—C10—H10C	109.5
C1—N2—B1	125.41 (17)	H10A—C10—H10C	109.5
C4—N2—B1	125.30 (17)	H10B—C10—H10C	109.5
O1—N3—O2	123.0 (2)	C3—C11—H11A	109.5
O1—N3—C2	118.71 (18)	C3—C11—H11B	109.5
O2—N3—C2	118.29 (19)	H11A—C11—H11B	109.5
N2—C1—C2	106.78 (18)	C3—C11—H11C	109.5
N2—C1—C10	122.98 (19)	H11A—C11—H11C	109.5
C2—C1—C10	130.1 (2)	H11B—C11—H11C	109.5
C1—C2—C3	110.67 (19)	C5—C12—H12A	109.5
C1—C2—N3	123.69 (19)	C5—C12—H12B	109.5
C3—C2—N3	125.57 (19)	H12A—C12—H12B	109.5
C2—C3—C4	104.34 (18)	C5—C12—H12C	109.5
C2—C3—C11	125.6 (2)	H12A—C12—H12C	109.5
C4—C3—C11	129.8 (2)	H12B—C12—H12C	109.5
N2—C4—C3	108.91 (17)	C7—C13—H13A	109.5
N2—C4—C5	120.27 (18)	C7—C13—H13B	109.5
C3—C4—C5	130.80 (19)	H13A—C13—H13B	109.5
C6—C5—C4	120.20 (18)	C7—C13—H13C	109.5
C6—C5—C12	119.87 (19)	H13A—C13—H13C	109.5
C4—C5—C12	119.91 (18)	H13B—C13—H13C	109.5
C5—C6—N1	120.78 (18)	C9—C14—H14A	109.5
C5—C6—C7	131.91 (18)	C9—C14—H14B	109.5
N1—C6—C7	107.31 (17)	H14A—C14—H14B	109.5
C8—C7—C6	106.11 (17)	C9—C14—H14C	109.5
C8—C7—C13	124.27 (19)	H14A—C14—H14C	109.5
C6—C7—C13	129.57 (19)	H14B—C14—H14C	109.5
C7—C8—C9	109.13 (19)	F1—B1—F2	109.40 (17)
C7—C8—H8A	125.4	F1—B1—N1	110.37 (18)
C9—C8—H8A	125.4	F2—B1—N1	110.00 (17)
N1—C9—C8	109.12 (18)	F1—B1—N2	110.71 (17)
N1—C9—C14	123.11 (18)	F2—B1—N2	109.91 (18)
C8—C9—C14	127.73 (19)	N1—B1—N2	106.41 (16)
			
C4—N2—C1—C2	−0.9 (2)	C4—C5—C6—C7	−174.0 (2)
B1—N2—C1—C2	179.69 (18)	C12—C5—C6—C7	7.7 (3)
C4—N2—C1—C10	175.32 (19)	C9—N1—C6—C5	178.89 (19)
B1—N2—C1—C10	−4.1 (3)	B1—N1—C6—C5	6.0 (3)
N2—C1—C2—C3	1.0 (2)	C9—N1—C6—C7	−1.5 (2)
C10—C1—C2—C3	−174.9 (2)	B1—N1—C6—C7	−174.36 (18)
N2—C1—C2—N3	−176.23 (19)	C5—C6—C7—C8	−179.9 (2)
C10—C1—C2—N3	7.9 (4)	N1—C6—C7—C8	0.5 (2)
O1—N3—C2—C1	22.4 (3)	C5—C6—C7—C13	2.5 (4)
O2—N3—C2—C1	−158.0 (2)	N1—C6—C7—C13	−177.1 (2)
O1—N3—C2—C3	−154.4 (2)	C6—C7—C8—C9	0.6 (2)
O2—N3—C2—C3	25.1 (3)	C13—C7—C8—C9	178.35 (19)
C1—C2—C3—C4	−0.7 (2)	C6—N1—C9—C8	1.9 (2)
N3—C2—C3—C4	176.5 (2)	B1—N1—C9—C8	174.73 (19)
C1—C2—C3—C11	−175.5 (2)	C6—N1—C9—C14	−175.81 (19)
N3—C2—C3—C11	1.7 (3)	B1—N1—C9—C14	−3.0 (3)
C1—N2—C4—C3	0.5 (2)	C7—C8—C9—N1	−1.6 (2)
B1—N2—C4—C3	179.93 (18)	C7—C8—C9—C14	176.0 (2)
C1—N2—C4—C5	179.00 (18)	C9—N1—B1—F1	−64.5 (3)
B1—N2—C4—C5	−1.6 (3)	C6—N1—B1—F1	107.2 (2)
C2—C3—C4—N2	0.1 (2)	C9—N1—B1—F2	56.3 (3)
C11—C3—C4—N2	174.6 (2)	C6—N1—B1—F2	−132.0 (2)
C2—C3—C4—C5	−178.2 (2)	C9—N1—B1—N2	175.34 (18)
C11—C3—C4—C5	−3.6 (4)	C6—N1—B1—N2	−13.0 (3)
N2—C4—C5—C6	−7.6 (3)	C1—N2—B1—F1	70.1 (3)
C3—C4—C5—C6	170.4 (2)	C4—N2—B1—F1	−109.2 (2)
N2—C4—C5—C12	170.72 (18)	C1—N2—B1—F2	−50.9 (3)
C3—C4—C5—C12	−11.2 (3)	C4—N2—B1—F2	129.82 (19)
C4—C5—C6—N1	5.5 (3)	C1—N2—B1—N1	−169.97 (18)
C12—C5—C6—N1	−172.83 (19)	C4—N2—B1—N1	10.8 (3)

### 4,4-Difluoro-1,3,5,7,8-pentamethyl-2-nitro-4-bora-3a,4a-diaza-s-indacene (5a) Hydrogen-bond geometry (Å, º)

**Table:**

*D*—H···*A*	*D*—H	H···*A*	*D*···*A*	*D*—H···*A*
C8—H8*A*···O2^i^	0.95	2.46	3.290 (3)	146
C10—H10*C*···O1^ii^	0.98	2.46	3.371 (3)	155
C12—H12*B*···F2^iii^	0.98	2.53	3.329 (3)	139

Symmetry codes: (i) *x*−1, *y*−1, *z*−1; (ii) −*x*+2, −*y*+1, −*z*+2; (iii) −*x*+1, −*y*+1, −*z*+1.

### 4,4-Difluoro-3-nitro-8-phenyl-4-bora-3a,4a-diaza-s-indacene (5b) Crystal data

**Table:**

C_15_H_10_BF_2_N_3_O_2_	*Z* = 2
*M**_r_* = 313.07	*F*(000) = 320
Triclinic, *P*1	*D*_x_ = 1.498 Mg m^−^^3^
*a* = 7.2833 (2) Å	Cu *K*α radiation, λ = 1.54178 Å
*b* = 8.5450 (3) Å	Cell parameters from 8868 reflections
*c* = 11.8803 (4) Å	θ = 3.9–67.0°
α = 81.093 (2)°	µ = 1.01 mm^−^^1^
β = 74.358 (2)°	*T* = 150 K
γ = 78.581 (2)°	Plate, orange
*V* = 693.86 (4) Å^3^	0.12 × 0.08 × 0.03 mm

### 4,4-Difluoro-3-nitro-8-phenyl-4-bora-3a,4a-diaza-s-indacene (5b) Data collection

**Table:**

Bruker Kappa APEX-DUO CCD diffractometer	2109 reflections with *I* > 2σ(*I*)
Radiation source: Bruker ImuS with multi-layer optics	*R*_int_ = 0.042
φ and ω scans	θ_max_ = 67.2°, θ_min_ = 3.9°
Absorption correction: multi-scan (SADABS, Bruker, 2014)	*h* = −8→8
*T*_min_ = 0.661, *T*_max_ = 0.753	*k* = −9→9
21668 measured reflections	*l* = −14→14
2453 independent reflections	

### 4,4-Difluoro-3-nitro-8-phenyl-4-bora-3a,4a-diaza-s-indacene (5b) Refinement

**Table:**

Refinement on *F*^2^	0 restraints
Least-squares matrix: full	Hydrogen site location: inferred from neighbouring sites
*R*[*F*^2^ > 2σ(*F*^2^)] = 0.033	H-atom parameters constrained
*wR*(*F*^2^) = 0.086	*w* = 1/[σ^2^(*F*_o_^2^) + (0.0461*P*)^2^ + 0.2263*P*] where *P* = (*F*_o_^2^ + 2*F*_c_^2^)/3
*S* = 1.05	(Δ/σ)_max_ < 0.001
2453 reflections	Δρ_max_ = 0.14 e Å^−^^3^
208 parameters	Δρ_min_ = −0.24 e Å^−^^3^

### 4,4-Difluoro-3-nitro-8-phenyl-4-bora-3a,4a-diaza-s-indacene (5b) Special details

**Table:**

Geometry. All esds (except the esd in the dihedral angle between two l.s. planes) are estimated using the full covariance matrix. The cell esds are taken into account individually in the estimation of esds in distances, angles and torsion angles; correlations between esds in cell parameters are only used when they are defined by crystal symmetry. An approximate (isotropic) treatment of cell esds is used for estimating esds involving l.s. planes.

### 4,4-Difluoro-3-nitro-8-phenyl-4-bora-3a,4a-diaza-s-indacene (5b) Fractional atomic coordinates and isotropic or equivalent isotropic displacement parameters (Å^2^)

**Table:**

	*x*	*y*	*z*	*U*_iso_*/*U*_eq_	
F1	0.86032 (11)	0.70745 (10)	0.51598 (7)	0.0232 (2)	
F2	0.77242 (12)	0.51313 (10)	0.44006 (7)	0.0271 (2)	
O1	0.80576 (16)	0.78510 (15)	0.28639 (10)	0.0377 (3)	
O2	0.58932 (16)	0.95022 (14)	0.21411 (9)	0.0339 (3)	
N1	0.65260 (16)	0.53533 (14)	0.64532 (10)	0.0212 (3)	
N2	0.52944 (16)	0.74478 (14)	0.50034 (10)	0.0182 (3)	
N3	0.64204 (18)	0.85969 (15)	0.29516 (10)	0.0232 (3)	
C1	0.4997 (2)	0.84792 (17)	0.40503 (12)	0.0192 (3)	
C2	0.3211 (2)	0.94448 (17)	0.42748 (13)	0.0218 (3)	
H2A	0.268753	1.023807	0.374059	0.026*	
C3	0.2339 (2)	0.90177 (17)	0.54401 (12)	0.0204 (3)	
H3A	0.109960	0.948262	0.586527	0.024*	
C4	0.36123 (19)	0.77789 (17)	0.58787 (12)	0.0183 (3)	
C5	0.3347 (2)	0.69070 (17)	0.70204 (12)	0.0191 (3)	
C6	0.4781 (2)	0.57103 (18)	0.72872 (12)	0.0212 (3)	
C7	0.4826 (2)	0.45638 (19)	0.82914 (13)	0.0282 (4)	
H7A	0.383249	0.450623	0.899664	0.034*	
C8	0.6557 (2)	0.3567 (2)	0.80526 (14)	0.0335 (4)	
H8A	0.699713	0.268516	0.856070	0.040*	
C9	0.7574 (2)	0.40853 (19)	0.69102 (14)	0.0285 (4)	
H9A	0.882959	0.359803	0.652302	0.034*	
C10	0.1497 (2)	0.72820 (17)	0.79003 (12)	0.0204 (3)	
C11	0.1464 (2)	0.7666 (2)	0.90056 (13)	0.0280 (4)	
H11A	0.264368	0.765447	0.920678	0.034*	
C12	−0.0275 (2)	0.8063 (2)	0.98091 (14)	0.0333 (4)	
H12A	−0.028448	0.832137	1.056029	0.040*	
C13	−0.2001 (2)	0.8085 (2)	0.95249 (14)	0.0314 (4)	
H13A	−0.319480	0.838283	1.007220	0.038*	
C14	−0.1983 (2)	0.7672 (2)	0.84422 (14)	0.0290 (4)	
H14A	−0.316824	0.766233	0.825435	0.035*	
C15	−0.0249 (2)	0.72708 (18)	0.76279 (13)	0.0243 (3)	
H15A	−0.024785	0.698866	0.688511	0.029*	
B1	0.7137 (2)	0.62463 (19)	0.51937 (14)	0.0195 (3)	

### 4,4-Difluoro-3-nitro-8-phenyl-4-bora-3a,4a-diaza-s-indacene (5b) Atomic displacement parameters (Å^2^)

**Table:**

	*U*^11^	*U*^22^	*U*^33^	*U*^12^	*U*^13^	*U*^23^
F1	0.0171 (4)	0.0269 (5)	0.0255 (4)	−0.0043 (3)	−0.0043 (3)	−0.0031 (3)
F2	0.0288 (5)	0.0252 (5)	0.0261 (5)	0.0018 (3)	−0.0049 (4)	−0.0107 (4)
O1	0.0238 (6)	0.0516 (8)	0.0253 (6)	0.0076 (5)	0.0023 (5)	0.0010 (5)
O2	0.0361 (6)	0.0390 (7)	0.0196 (5)	−0.0002 (5)	−0.0043 (5)	0.0056 (5)
N1	0.0178 (6)	0.0225 (6)	0.0224 (6)	−0.0020 (5)	−0.0048 (5)	−0.0018 (5)
N2	0.0167 (6)	0.0210 (6)	0.0163 (6)	−0.0031 (5)	−0.0023 (5)	−0.0038 (5)
N3	0.0250 (7)	0.0261 (7)	0.0170 (6)	−0.0040 (5)	−0.0025 (5)	−0.0026 (5)
C1	0.0210 (7)	0.0216 (7)	0.0152 (7)	−0.0049 (6)	−0.0034 (5)	−0.0020 (5)
C2	0.0215 (7)	0.0213 (7)	0.0225 (7)	−0.0022 (6)	−0.0070 (6)	−0.0008 (6)
C3	0.0167 (7)	0.0230 (8)	0.0200 (7)	−0.0020 (5)	−0.0023 (5)	−0.0037 (6)
C4	0.0157 (7)	0.0214 (7)	0.0182 (7)	−0.0042 (5)	−0.0019 (5)	−0.0056 (5)
C5	0.0191 (7)	0.0221 (8)	0.0179 (7)	−0.0064 (6)	−0.0039 (5)	−0.0046 (6)
C6	0.0197 (7)	0.0252 (8)	0.0185 (7)	−0.0061 (6)	−0.0026 (6)	−0.0023 (6)
C7	0.0260 (8)	0.0323 (9)	0.0238 (8)	−0.0060 (6)	−0.0045 (6)	0.0037 (6)
C8	0.0325 (9)	0.0330 (9)	0.0301 (9)	−0.0007 (7)	−0.0095 (7)	0.0086 (7)
C9	0.0223 (8)	0.0285 (8)	0.0314 (8)	0.0007 (6)	−0.0075 (6)	0.0014 (7)
C10	0.0203 (7)	0.0211 (7)	0.0179 (7)	−0.0043 (6)	−0.0013 (6)	−0.0016 (5)
C11	0.0268 (8)	0.0384 (9)	0.0197 (7)	−0.0086 (7)	−0.0045 (6)	−0.0044 (6)
C12	0.0382 (9)	0.0419 (10)	0.0189 (8)	−0.0110 (7)	0.0006 (7)	−0.0081 (7)
C13	0.0271 (8)	0.0340 (9)	0.0256 (8)	−0.0048 (7)	0.0061 (6)	−0.0038 (7)
C14	0.0193 (7)	0.0349 (9)	0.0297 (8)	−0.0043 (6)	−0.0022 (6)	−0.0014 (7)
C15	0.0224 (7)	0.0292 (8)	0.0210 (7)	−0.0059 (6)	−0.0033 (6)	−0.0035 (6)
B1	0.0182 (8)	0.0203 (8)	0.0189 (8)	−0.0014 (6)	−0.0026 (6)	−0.0045 (6)

### 4,4-Difluoro-3-nitro-8-phenyl-4-bora-3a,4a-diaza-s-indacene (5b) Geometric parameters (Å, º)

**Table:**

F1—B1	1.3822 (18)	C5—C10	1.4782 (19)
F2—B1	1.3703 (18)	C6—C7	1.425 (2)
O1—N3	1.2213 (16)	C7—C8	1.361 (2)
O2—N3	1.2346 (16)	C7—H7A	0.9500
N1—C9	1.3288 (19)	C8—C9	1.410 (2)
N1—C6	1.3970 (18)	C8—H8A	0.9500
N1—B1	1.5623 (19)	C9—H9A	0.9500
N2—C1	1.3614 (18)	C10—C11	1.395 (2)
N2—C4	1.3889 (17)	C10—C15	1.396 (2)
N2—B1	1.5659 (19)	C11—C12	1.381 (2)
N3—C1	1.4351 (18)	C11—H11A	0.9500
C1—C2	1.379 (2)	C12—C13	1.383 (2)
C2—C3	1.384 (2)	C12—H12A	0.9500
C2—H2A	0.9500	C13—C14	1.382 (2)
C3—C4	1.397 (2)	C13—H13A	0.9500
C3—H3A	0.9500	C14—C15	1.386 (2)
C4—C5	1.427 (2)	C14—H14A	0.9500
C5—C6	1.375 (2)	C15—H15A	0.9500
			
C9—N1—C6	107.79 (12)	C7—C8—C9	107.24 (14)
C9—N1—B1	125.56 (12)	C7—C8—H8A	126.4
C6—N1—B1	126.63 (11)	C9—C8—H8A	126.4
C1—N2—C4	104.81 (11)	N1—C9—C8	110.26 (14)
C1—N2—B1	130.53 (12)	N1—C9—H9A	124.9
C4—N2—B1	124.41 (11)	C8—C9—H9A	124.9
O1—N3—O2	123.67 (12)	C11—C10—C15	119.09 (13)
O1—N3—C1	120.11 (12)	C11—C10—C5	120.78 (13)
O2—N3—C1	116.21 (12)	C15—C10—C5	120.13 (12)
N2—C1—C2	112.49 (12)	C12—C11—C10	120.37 (14)
N2—C1—N3	123.66 (12)	C12—C11—H11A	119.8
C2—C1—N3	123.79 (13)	C10—C11—H11A	119.8
C1—C2—C3	105.65 (12)	C11—C12—C13	120.30 (14)
C1—C2—H2A	127.2	C11—C12—H12A	119.9
C3—C2—H2A	127.2	C13—C12—H12A	119.9
C2—C3—C4	107.64 (12)	C14—C13—C12	119.76 (14)
C2—C3—H3A	126.2	C14—C13—H13A	120.1
C4—C3—H3A	126.2	C12—C13—H13A	120.1
N2—C4—C3	109.40 (12)	C13—C14—C15	120.52 (14)
N2—C4—C5	121.78 (12)	C13—C14—H14A	119.7
C3—C4—C5	128.82 (13)	C15—C14—H14A	119.7
C6—C5—C4	120.28 (13)	C14—C15—C10	119.94 (14)
C6—C5—C10	120.61 (13)	C14—C15—H15A	120.0
C4—C5—C10	119.09 (12)	C10—C15—H15A	120.0
C5—C6—N1	120.54 (13)	F2—B1—F1	111.50 (12)
C5—C6—C7	131.83 (14)	F2—B1—N1	108.45 (12)
N1—C6—C7	107.41 (12)	F1—B1—N1	108.76 (11)
C8—C7—C6	107.29 (14)	F2—B1—N2	111.91 (12)
C8—C7—H7A	126.4	F1—B1—N2	110.28 (12)
C6—C7—H7A	126.4	N1—B1—N2	105.71 (11)
			
C4—N2—C1—C2	−0.17 (16)	N1—C6—C7—C8	0.26 (17)
B1—N2—C1—C2	−174.36 (13)	C6—C7—C8—C9	−0.16 (19)
C4—N2—C1—N3	177.18 (12)	C6—N1—C9—C8	0.16 (18)
B1—N2—C1—N3	3.0 (2)	B1—N1—C9—C8	−178.48 (14)
O1—N3—C1—N2	−6.6 (2)	C7—C8—C9—N1	0.0 (2)
O2—N3—C1—N2	174.85 (13)	C6—C5—C10—C11	55.5 (2)
O1—N3—C1—C2	170.45 (14)	C4—C5—C10—C11	−125.98 (15)
O2—N3—C1—C2	−8.1 (2)	C6—C5—C10—C15	−125.16 (15)
N2—C1—C2—C3	0.92 (16)	C4—C5—C10—C15	53.40 (19)
N3—C1—C2—C3	−176.43 (13)	C15—C10—C11—C12	−1.4 (2)
C1—C2—C3—C4	−1.28 (16)	C5—C10—C11—C12	178.01 (14)
C1—N2—C4—C3	−0.65 (15)	C10—C11—C12—C13	−0.1 (3)
B1—N2—C4—C3	173.99 (12)	C11—C12—C13—C14	1.5 (3)
C1—N2—C4—C5	178.59 (12)	C12—C13—C14—C15	−1.5 (2)
B1—N2—C4—C5	−6.8 (2)	C13—C14—C15—C10	0.0 (2)
C2—C3—C4—N2	1.23 (16)	C11—C10—C15—C14	1.4 (2)
C2—C3—C4—C5	−177.94 (14)	C5—C10—C15—C14	−178.00 (14)
N2—C4—C5—C6	0.6 (2)	C9—N1—B1—F2	50.52 (18)
C3—C4—C5—C6	179.67 (14)	C6—N1—B1—F2	−127.86 (14)
N2—C4—C5—C10	−177.97 (12)	C9—N1—B1—F1	−70.91 (17)
C3—C4—C5—C10	1.1 (2)	C6—N1—B1—F1	110.71 (14)
C4—C5—C6—N1	1.2 (2)	C9—N1—B1—N2	170.68 (13)
C10—C5—C6—N1	179.75 (12)	C6—N1—B1—N2	−7.70 (18)
C4—C5—C6—C7	−172.63 (15)	C1—N2—B1—F2	−59.63 (19)
C10—C5—C6—C7	5.9 (2)	C4—N2—B1—F2	127.19 (13)
C9—N1—C6—C5	−175.45 (13)	C1—N2—B1—F1	65.10 (18)
B1—N1—C6—C5	3.2 (2)	C4—N2—B1—F1	−108.08 (14)
C9—N1—C6—C7	−0.25 (16)	C1—N2—B1—N1	−177.50 (13)
B1—N1—C6—C7	178.36 (13)	C4—N2—B1—N1	9.32 (17)
C5—C6—C7—C8	174.70 (16)		

### 4,4-Difluoro-3-nitro-8-phenyl-4-bora-3a,4a-diaza-s-indacene (5b) Hydrogen-bond geometry (Å, º)

**Table:**

*D*—H···*A*	*D*—H	H···*A*	*D*···*A*	*D*—H···*A*
C9—H9*A*···F1^i^	0.95	2.40	3.2788 (18)	155
C9—H9*A*···O1^i^	0.95	2.59	3.3420 (19)	136
C15—H15*A*···F1^ii^	0.95	2.40	3.2946 (17)	157

Symmetry codes: (i) −*x*+2, −*y*+1, −*z*+1; (ii) *x*−1, *y*, *z*.

### 3-Chloro-6-ethyl-5,7,8-trimethyl-2-nitro-4,4-diphenyl-4-bora-3a,4a-diaza-s-indacene (5d) Crystal data

**Table:**

C_26_H_25_BClN_3_O_2_	*F*(000) = 960
*M**_r_* = 457.75	*D*_x_ = 1.322 Mg m^−^^3^
Monoclinic, *P*2_1_/*n*	Cu *K*α radiation, λ = 1.54178 Å
*a* = 11.8359 (4) Å	Cell parameters from 9143 reflections
*b* = 12.0825 (4) Å	θ = 4.2–66.9°
*c* = 16.5811 (5) Å	µ = 1.70 mm^−^^1^
β = 104.116 (1)°	*T* = 150 K
*V* = 2299.62 (13) Å^3^	Block, orange
*Z* = 4	0.19 × 0.18 × 0.10 mm

### 3-Chloro-6-ethyl-5,7,8-trimethyl-2-nitro-4,4-diphenyl-4-bora-3a,4a-diaza-s-indacene (5d) Data collection

**Table:**

Bruker Kappa APEX-DUO CCD diffractometer	3864 reflections with *I* > 2σ(*I*)
Radiation source: Bruker ImuS with multi-layer optics	*R*_int_ = 0.047
φ and ω scans	θ_max_ = 67.0°, θ_min_ = 4.2°
Absorption correction: multi-scan (SADABS, Bruker, 2014)	*h* = −14→14
*T*_min_ = 0.586, *T*_max_ = 0.753	*k* = −14→14
43708 measured reflections	*l* = −19→19
4080 independent reflections	

### 3-Chloro-6-ethyl-5,7,8-trimethyl-2-nitro-4,4-diphenyl-4-bora-3a,4a-diaza-s-indacene (5d) Refinement

**Table:**

Refinement on *F*^2^	8 restraints
Least-squares matrix: full	Hydrogen site location: inferred from neighbouring sites
*R*[*F*^2^ > 2σ(*F*^2^)] = 0.033	H-atom parameters constrained
*wR*(*F*^2^) = 0.085	*w* = 1/[σ^2^(*F*_o_^2^) + (0.037*P*)^2^ + 1.0066*P*] where *P* = (*F*_o_^2^ + 2*F*_c_^2^)/3
*S* = 1.04	(Δ/σ)_max_ = 0.002
4080 reflections	Δρ_max_ = 0.27 e Å^−^^3^
330 parameters	Δρ_min_ = −0.29 e Å^−^^3^

### 3-Chloro-6-ethyl-5,7,8-trimethyl-2-nitro-4,4-diphenyl-4-bora-3a,4a-diaza-s-indacene (5d) Special details

**Table:**

Geometry. All esds (except the esd in the dihedral angle between two l.s. planes) are estimated using the full covariance matrix. The cell esds are taken into account individually in the estimation of esds in distances, angles and torsion angles; correlations between esds in cell parameters are only used when they are defined by crystal symmetry. An approximate (isotropic) treatment of cell esds is used for estimating esds involving l.s. planes.

### 3-Chloro-6-ethyl-5,7,8-trimethyl-2-nitro-4,4-diphenyl-4-bora-3a,4a-diaza-s-indacene (5d) Fractional atomic coordinates and isotropic or equivalent isotropic displacement parameters (Å^2^)

**Table:**

	*x*	*y*	*z*	*U*_iso_*/*U*_eq_	Occ. (<1)
Cl1	0.65541 (3)	0.48753 (3)	0.73750 (2)	0.03002 (11)	
O1	0.8114 (8)	0.6549 (7)	0.8363 (6)	0.0496 (19)	0.618 (12)
O2	0.7942 (2)	0.7083 (3)	0.9601 (2)	0.0483 (9)	0.618 (12)
O1A	0.8229 (13)	0.6468 (11)	0.8446 (9)	0.047 (3)	0.382 (12)
O2A	0.7609 (8)	0.7616 (10)	0.9191 (9)	0.106 (5)	0.382 (12)
N3A	0.7493 (6)	0.6751 (7)	0.8782 (6)	0.057 (4)	0.382 (12)
N1	0.28379 (9)	0.44041 (9)	0.79566 (6)	0.0220 (2)	
N2	0.49001 (9)	0.50641 (8)	0.82271 (6)	0.0202 (2)	
N3	0.7575 (4)	0.6637 (4)	0.8919 (3)	0.0346 (13)	0.618 (12)
C1	0.59526 (11)	0.53714 (11)	0.81333 (8)	0.0230 (3)	
C2	0.64420 (12)	0.61408 (11)	0.87475 (9)	0.0282 (3)	
C3	0.56525 (12)	0.63145 (11)	0.92368 (9)	0.0289 (3)	
H3A	0.574961	0.679231	0.970370	0.035*	
C4	0.47007 (11)	0.56522 (10)	0.89058 (7)	0.0217 (3)	
C5	0.36080 (11)	0.56043 (10)	0.91296 (8)	0.0225 (3)	
C6	0.27018 (11)	0.50137 (10)	0.86548 (8)	0.0225 (3)	
C7	0.14980 (12)	0.49154 (11)	0.86915 (9)	0.0265 (3)	
C8	0.09486 (11)	0.42855 (11)	0.80284 (9)	0.0274 (3)	
C9	0.18088 (11)	0.39655 (11)	0.75961 (8)	0.0258 (3)	
C10	0.34888 (12)	0.62834 (12)	0.98638 (8)	0.0292 (3)	
H10A	0.309866	0.584389	1.021281	0.044*	
H10B	0.302660	0.694673	0.966808	0.044*	
H10C	0.426341	0.650229	1.018928	0.044*	
C11	0.09350 (13)	0.53934 (14)	0.93294 (10)	0.0363 (3)	
H11A	0.008965	0.528827	0.915024	0.054*	
H11B	0.111057	0.618591	0.939270	0.054*	
H11C	0.123598	0.501911	0.986295	0.054*	
C12	−0.03133 (12)	0.39544 (13)	0.77918 (10)	0.0355 (3)	
H12A	−0.054369	0.379843	0.718764	0.043*	
H12B	−0.079245	0.458094	0.790330	0.043*	
C13	−0.05679 (15)	0.29403 (16)	0.82626 (14)	0.0541 (5)	
H13A	−0.140105	0.276718	0.809033	0.081*	
H13B	−0.034929	0.309133	0.886132	0.081*	
H13C	−0.011725	0.230943	0.813942	0.081*	
C14	0.16000 (12)	0.32166 (13)	0.68649 (10)	0.0359 (3)	
H14A	0.222686	0.266743	0.694415	0.054*	
H14B	0.158343	0.364994	0.636252	0.054*	
H14C	0.085190	0.283795	0.680523	0.054*	
C15	0.39216 (10)	0.42485 (11)	0.67672 (8)	0.0232 (3)	
C16	0.41692 (13)	0.34137 (12)	0.62563 (8)	0.0319 (3)	
H16A	0.442040	0.271151	0.649036	0.038*	
C17	0.40564 (15)	0.35864 (15)	0.54121 (9)	0.0431 (4)	
H17A	0.422943	0.300438	0.507608	0.052*	
C18	0.36943 (14)	0.45996 (17)	0.50602 (9)	0.0456 (4)	
H18A	0.361863	0.471750	0.448300	0.055*	
C19	0.34425 (14)	0.54409 (15)	0.55507 (10)	0.0430 (4)	
H19A	0.319276	0.614100	0.531195	0.052*	
C20	0.35542 (12)	0.52636 (13)	0.63928 (9)	0.0317 (3)	
H20A	0.337577	0.584903	0.672367	0.038*	
C21	0.44966 (11)	0.29287 (10)	0.81776 (7)	0.0218 (3)	
C22	0.39015 (11)	0.23405 (11)	0.86739 (8)	0.0263 (3)	
H22A	0.317671	0.261679	0.873739	0.032*	
C23	0.43379 (13)	0.13640 (12)	0.90770 (9)	0.0336 (3)	
H23A	0.390919	0.098571	0.940763	0.040*	
C24	0.53879 (14)	0.09428 (12)	0.89995 (10)	0.0364 (3)	
H24A	0.569302	0.028327	0.928206	0.044*	
C25	0.59920 (13)	0.14938 (12)	0.85042 (10)	0.0358 (3)	
H25A	0.671213	0.120649	0.844002	0.043*	
C26	0.55507 (12)	0.24623 (11)	0.81020 (8)	0.0291 (3)	
H26A	0.597767	0.282408	0.776227	0.035*	
B1	0.40435 (12)	0.41142 (12)	0.77529 (9)	0.0205 (3)	

### 3-Chloro-6-ethyl-5,7,8-trimethyl-2-nitro-4,4-diphenyl-4-bora-3a,4a-diaza-s-indacene (5d) Atomic displacement parameters (Å^2^)

**Table:**

	*U*^11^	*U*^22^	*U*^33^	*U*^12^	*U*^13^	*U*^23^
Cl1	0.02361 (17)	0.0383 (2)	0.03203 (19)	−0.00318 (12)	0.01435 (13)	−0.00588 (13)
O1	0.029 (3)	0.076 (4)	0.051 (2)	−0.023 (2)	0.024 (3)	−0.011 (2)
O2	0.0407 (12)	0.0506 (17)	0.0490 (17)	−0.0191 (11)	0.0021 (11)	−0.0194 (13)
O1A	0.027 (4)	0.039 (4)	0.071 (7)	−0.002 (2)	0.007 (3)	−0.015 (3)
O2A	0.083 (5)	0.130 (8)	0.129 (8)	−0.078 (5)	0.068 (6)	−0.101 (7)
N3A	0.045 (5)	0.082 (8)	0.045 (4)	−0.037 (4)	0.016 (3)	−0.042 (4)
N1	0.0208 (5)	0.0231 (5)	0.0231 (5)	−0.0005 (4)	0.0074 (4)	−0.0010 (4)
N2	0.0194 (5)	0.0214 (5)	0.0203 (5)	0.0007 (4)	0.0062 (4)	−0.0003 (4)
N3	0.024 (2)	0.0271 (17)	0.052 (3)	−0.0026 (14)	0.0089 (15)	−0.005 (2)
C1	0.0200 (6)	0.0253 (6)	0.0246 (6)	0.0008 (5)	0.0072 (5)	0.0010 (5)
C2	0.0228 (7)	0.0292 (7)	0.0329 (7)	−0.0050 (5)	0.0070 (5)	−0.0041 (6)
C3	0.0290 (7)	0.0286 (7)	0.0287 (7)	−0.0019 (5)	0.0065 (6)	−0.0084 (5)
C4	0.0241 (6)	0.0218 (6)	0.0196 (6)	0.0027 (5)	0.0059 (5)	−0.0006 (5)
C5	0.0253 (6)	0.0219 (6)	0.0214 (6)	0.0048 (5)	0.0079 (5)	0.0027 (5)
C6	0.0236 (6)	0.0233 (6)	0.0227 (6)	0.0038 (5)	0.0099 (5)	0.0025 (5)
C7	0.0247 (7)	0.0260 (6)	0.0317 (7)	0.0042 (5)	0.0127 (6)	0.0063 (5)
C8	0.0212 (6)	0.0281 (7)	0.0345 (7)	0.0015 (5)	0.0097 (5)	0.0066 (6)
C9	0.0217 (6)	0.0263 (7)	0.0298 (7)	−0.0013 (5)	0.0069 (5)	0.0014 (5)
C10	0.0312 (7)	0.0338 (7)	0.0243 (7)	0.0041 (6)	0.0104 (6)	−0.0033 (6)
C11	0.0311 (8)	0.0439 (8)	0.0403 (8)	0.0058 (6)	0.0210 (7)	0.0033 (7)
C12	0.0219 (7)	0.0378 (8)	0.0484 (9)	−0.0001 (6)	0.0115 (6)	0.0043 (7)
C13	0.0376 (9)	0.0475 (10)	0.0789 (13)	−0.0111 (8)	0.0178 (9)	0.0141 (9)
C14	0.0254 (7)	0.0410 (8)	0.0409 (8)	−0.0087 (6)	0.0074 (6)	−0.0121 (7)
C15	0.0178 (6)	0.0301 (7)	0.0220 (6)	−0.0041 (5)	0.0056 (5)	0.0001 (5)
C16	0.0395 (8)	0.0342 (7)	0.0241 (7)	−0.0076 (6)	0.0118 (6)	−0.0042 (6)
C17	0.0520 (10)	0.0558 (10)	0.0253 (7)	−0.0184 (8)	0.0169 (7)	−0.0093 (7)
C18	0.0392 (9)	0.0762 (12)	0.0202 (7)	−0.0208 (8)	0.0052 (6)	0.0075 (8)
C19	0.0325 (8)	0.0565 (10)	0.0376 (9)	−0.0019 (7)	0.0037 (6)	0.0217 (8)
C20	0.0249 (7)	0.0376 (8)	0.0324 (7)	0.0020 (6)	0.0063 (6)	0.0069 (6)
C21	0.0242 (6)	0.0229 (6)	0.0183 (6)	−0.0017 (5)	0.0055 (5)	−0.0038 (5)
C22	0.0245 (6)	0.0280 (7)	0.0269 (7)	−0.0024 (5)	0.0071 (5)	−0.0002 (5)
C23	0.0373 (8)	0.0314 (7)	0.0323 (7)	−0.0060 (6)	0.0086 (6)	0.0065 (6)
C24	0.0431 (9)	0.0266 (7)	0.0376 (8)	0.0053 (6)	0.0063 (7)	0.0069 (6)
C25	0.0359 (8)	0.0332 (8)	0.0402 (8)	0.0108 (6)	0.0131 (6)	0.0020 (6)
C26	0.0318 (7)	0.0284 (7)	0.0305 (7)	0.0039 (6)	0.0142 (6)	0.0017 (6)
B1	0.0185 (7)	0.0224 (7)	0.0219 (7)	−0.0013 (5)	0.0075 (5)	−0.0028 (5)

### 3-Chloro-6-ethyl-5,7,8-trimethyl-2-nitro-4,4-diphenyl-4-bora-3a,4a-diaza-s-indacene (5d) Geometric parameters (Å, º)

**Table:**

Cl1—C1	1.6982 (13)	C12—H12A	0.9900
O1—N3	1.248 (6)	C12—H12B	0.9900
O2—N3	1.232 (5)	C13—H13A	0.9800
O1A—N3A	1.193 (12)	C13—H13B	0.9800
O2A—N3A	1.235 (12)	C13—H13C	0.9800
N3A—C2	1.435 (7)	C14—H14A	0.9800
N1—C9	1.3292 (17)	C14—H14B	0.9800
N1—C6	1.4140 (16)	C14—H14C	0.9800
N1—B1	1.5836 (16)	C15—C16	1.3935 (19)
N2—C1	1.3447 (17)	C15—C20	1.395 (2)
N2—C4	1.3989 (16)	C15—B1	1.6134 (18)
N2—B1	1.6045 (17)	C16—C17	1.389 (2)
N3—C2	1.433 (4)	C16—H16A	0.9500
C1—C2	1.3962 (19)	C17—C18	1.379 (3)
C2—C3	1.3947 (19)	C17—H17A	0.9500
C3—C4	1.3817 (19)	C18—C19	1.379 (3)
C3—H3A	0.9500	C18—H18A	0.9500
C4—C5	1.4309 (18)	C19—C20	1.387 (2)
C5—C6	1.3666 (19)	C19—H19A	0.9500
C5—C10	1.5030 (17)	C20—H20A	0.9500
C6—C7	1.4457 (19)	C21—C22	1.4003 (18)
C7—C8	1.365 (2)	C21—C26	1.4022 (19)
C7—C11	1.4968 (19)	C21—B1	1.6283 (18)
C8—C9	1.4339 (19)	C22—C23	1.392 (2)
C8—C12	1.5030 (19)	C22—H22A	0.9500
C9—C14	1.4845 (19)	C23—C24	1.378 (2)
C10—H10A	0.9800	C23—H23A	0.9500
C10—H10B	0.9800	C24—C25	1.384 (2)
C10—H10C	0.9800	C24—H24A	0.9500
C11—H11A	0.9800	C25—C26	1.385 (2)
C11—H11B	0.9800	C25—H25A	0.9500
C11—H11C	0.9800	C26—H26A	0.9500
C12—C13	1.522 (2)		
			
O1A—N3A—O2A	120.2 (9)	C13—C12—H12A	109.0
O1A—N3A—C2	124.0 (10)	C8—C12—H12B	109.0
O2A—N3A—C2	115.8 (7)	C13—C12—H12B	109.0
C9—N1—C6	107.52 (10)	H12A—C12—H12B	107.8
C9—N1—B1	126.18 (11)	C12—C13—H13A	109.5
C6—N1—B1	125.33 (10)	C12—C13—H13B	109.5
C1—N2—C4	107.19 (10)	H13A—C13—H13B	109.5
C1—N2—B1	129.30 (10)	C12—C13—H13C	109.5
C4—N2—B1	123.17 (10)	H13A—C13—H13C	109.5
O2—N3—O1	125.9 (6)	H13B—C13—H13C	109.5
O1A—N3—O2A	114.0 (7)	C9—C14—H14A	109.5
O2—N3—C2	117.9 (3)	C9—C14—H14B	109.5
O1A—N3—C2	120.2 (8)	H14A—C14—H14B	109.5
O1—N3—C2	116.2 (5)	C9—C14—H14C	109.5
O2A—N3—C2	114.1 (4)	H14A—C14—H14C	109.5
N2—C1—C2	109.32 (11)	H14B—C14—H14C	109.5
N2—C1—Cl1	123.75 (10)	C16—C15—C20	117.04 (12)
C2—C1—Cl1	126.93 (10)	C16—C15—B1	124.26 (12)
C3—C2—C1	107.87 (12)	C20—C15—B1	118.70 (12)
C3—C2—N3	123.3 (2)	C17—C16—C15	121.41 (15)
C1—C2—N3	128.7 (2)	C17—C16—H16A	119.3
C3—C2—N3A	126.6 (4)	C15—C16—H16A	119.3
C1—C2—N3A	124.9 (4)	C18—C17—C16	120.23 (16)
C4—C3—C2	106.19 (12)	C18—C17—H17A	119.9
C4—C3—H3A	126.9	C16—C17—H17A	119.9
C2—C3—H3A	126.9	C17—C18—C19	119.64 (14)
C3—C4—N2	109.42 (11)	C17—C18—H18A	120.2
C3—C4—C5	128.41 (12)	C19—C18—H18A	120.2
N2—C4—C5	121.91 (11)	C18—C19—C20	119.91 (15)
C6—C5—C4	120.25 (11)	C18—C19—H19A	120.0
C6—C5—C10	122.42 (12)	C20—C19—H19A	120.0
C4—C5—C10	117.21 (11)	C19—C20—C15	121.78 (15)
C5—C6—N1	120.90 (11)	C19—C20—H20A	119.1
C5—C6—C7	131.43 (12)	C15—C20—H20A	119.1
N1—C6—C7	107.57 (11)	C22—C21—C26	115.76 (12)
C8—C7—C6	107.02 (12)	C22—C21—B1	122.74 (11)
C8—C7—C11	125.29 (13)	C26—C21—B1	121.41 (11)
C6—C7—C11	127.68 (13)	C23—C22—C21	122.11 (13)
C7—C8—C9	107.23 (12)	C23—C22—H22A	118.9
C7—C8—C12	127.35 (13)	C21—C22—H22A	118.9
C9—C8—C12	125.40 (13)	C24—C23—C22	120.39 (13)
N1—C9—C8	110.62 (12)	C24—C23—H23A	119.8
N1—C9—C14	124.21 (12)	C22—C23—H23A	119.8
C8—C9—C14	125.14 (12)	C23—C24—C25	119.09 (13)
C5—C10—H10A	109.5	C23—C24—H24A	120.5
C5—C10—H10B	109.5	C25—C24—H24A	120.5
H10A—C10—H10B	109.5	C24—C25—C26	120.24 (13)
C5—C10—H10C	109.5	C24—C25—H25A	119.9
H10A—C10—H10C	109.5	C26—C25—H25A	119.9
H10B—C10—H10C	109.5	C25—C26—C21	122.40 (13)
C7—C11—H11A	109.5	C25—C26—H26A	118.8
C7—C11—H11B	109.5	C21—C26—H26A	118.8
H11A—C11—H11B	109.5	N1—B1—N2	103.40 (9)
C7—C11—H11C	109.5	N1—B1—C15	109.35 (10)
H11A—C11—H11C	109.5	N2—B1—C15	108.31 (10)
H11B—C11—H11C	109.5	N1—B1—C21	108.73 (10)
C8—C12—C13	112.89 (13)	N2—B1—C21	108.37 (10)
C8—C12—H12A	109.0	C15—B1—C21	117.72 (10)
			
N3—O1A—N3A—O2A	93 (2)	C5—C6—C7—C11	−5.2 (2)
N3—O1A—N3A—C2	−87 (2)	N1—C6—C7—C11	178.51 (13)
N3—O2A—N3A—O1A	−96 (2)	C6—C7—C8—C9	1.71 (14)
N3—O2A—N3A—C2	84 (2)	C11—C7—C8—C9	−177.82 (13)
N3A—O1A—N3—O2A	−67 (3)	C6—C7—C8—C12	−179.59 (13)
N3A—O1A—N3—C2	73 (2)	C11—C7—C8—C12	0.9 (2)
N3A—O2A—N3—O1A	64 (3)	C6—N1—C9—C8	1.26 (14)
N3A—O2A—N3—C2	−79 (2)	B1—N1—C9—C8	170.46 (11)
C4—N2—C1—C2	0.65 (14)	C6—N1—C9—C14	−176.74 (13)
B1—N2—C1—C2	−172.70 (12)	B1—N1—C9—C14	−7.5 (2)
C4—N2—C1—Cl1	−179.37 (9)	C7—C8—C9—N1	−1.92 (15)
B1—N2—C1—Cl1	7.27 (18)	C12—C8—C9—N1	179.35 (12)
N2—C1—C2—C3	−0.21 (16)	C7—C8—C9—C14	176.06 (13)
Cl1—C1—C2—C3	179.82 (10)	C12—C8—C9—C14	−2.7 (2)
N2—C1—C2—N3	175.6 (3)	C7—C8—C12—C13	−83.32 (19)
Cl1—C1—C2—N3	−4.3 (4)	C9—C8—C12—C13	95.16 (18)
N2—C1—C2—N3A	−172.0 (4)	C20—C15—C16—C17	0.1 (2)
Cl1—C1—C2—N3A	8.0 (4)	B1—C15—C16—C17	−179.39 (13)
O2—N3—C2—C3	12.5 (6)	C15—C16—C17—C18	0.1 (2)
O1A—N3—C2—C3	−178.3 (10)	C16—C17—C18—C19	−0.1 (2)
O1—N3—C2—C3	−169.2 (6)	C17—C18—C19—C20	0.0 (2)
O2A—N3—C2—C3	−37.4 (9)	C18—C19—C20—C15	0.2 (2)
O2—N3—C2—C1	−162.8 (3)	C16—C15—C20—C19	−0.2 (2)
O1A—N3—C2—C1	6.4 (12)	B1—C15—C20—C19	179.29 (13)
O1—N3—C2—C1	15.5 (8)	C26—C21—C22—C23	0.98 (19)
O2A—N3—C2—C1	147.3 (8)	B1—C21—C22—C23	−175.73 (12)
O1A—N3—C2—N3A	−66 (3)	C21—C22—C23—C24	0.2 (2)
O2A—N3—C2—N3A	74 (3)	C22—C23—C24—C25	−1.1 (2)
O1A—N3A—C2—C3	167.9 (8)	C23—C24—C25—C26	0.8 (2)
O2A—N3A—C2—C3	−12.1 (8)	C24—C25—C26—C21	0.4 (2)
O1A—N3A—C2—C1	−21.8 (8)	C22—C21—C26—C25	−1.3 (2)
O2A—N3A—C2—C1	158.2 (8)	B1—C21—C26—C25	175.49 (12)
O1A—N3A—C2—N3	93 (3)	C9—N1—B1—N2	168.35 (11)
O2A—N3A—C2—N3	−87 (3)	C6—N1—B1—N2	−24.31 (15)
C1—C2—C3—C4	−0.32 (16)	C9—N1—B1—C15	53.14 (16)
N3—C2—C3—C4	−176.4 (3)	C6—N1—B1—C15	−139.52 (11)
N3A—C2—C3—C4	171.3 (4)	C9—N1—B1—C21	−76.62 (15)
C2—C3—C4—N2	0.72 (15)	C6—N1—B1—C21	90.71 (13)
C2—C3—C4—C5	−173.48 (13)	C1—N2—B1—N1	−164.39 (11)
C1—N2—C4—C3	−0.86 (14)	C4—N2—B1—N1	23.20 (14)
B1—N2—C4—C3	172.99 (11)	C1—N2—B1—C15	−48.44 (16)
C1—N2—C4—C5	173.79 (11)	C4—N2—B1—C15	139.15 (11)
B1—N2—C4—C5	−12.35 (17)	C1—N2—B1—C21	80.33 (15)
C3—C4—C5—C6	170.50 (13)	C4—N2—B1—C21	−92.08 (13)
N2—C4—C5—C6	−3.07 (18)	C16—C15—B1—N1	−124.11 (13)
C3—C4—C5—C10	−5.6 (2)	C20—C15—B1—N1	56.42 (15)
N2—C4—C5—C10	−179.18 (11)	C16—C15—B1—N2	123.87 (13)
C4—C5—C6—N1	2.62 (18)	C20—C15—B1—N2	−55.61 (14)
C10—C5—C6—N1	178.52 (11)	C16—C15—B1—C21	0.58 (18)
C4—C5—C6—C7	−173.26 (13)	C20—C15—B1—C21	−178.90 (11)
C10—C5—C6—C7	2.6 (2)	C22—C21—B1—N1	−3.28 (16)
C9—N1—C6—C5	−176.94 (12)	C26—C21—B1—N1	−179.80 (11)
B1—N1—C6—C5	13.75 (18)	C22—C21—B1—N2	108.47 (13)
C9—N1—C6—C7	−0.18 (14)	C26—C21—B1—N2	−68.05 (14)
B1—N1—C6—C7	−169.49 (11)	C22—C21—B1—C15	−128.27 (13)
C5—C6—C7—C8	175.29 (14)	C26—C21—B1—C15	55.21 (16)
N1—C6—C7—C8	−1.00 (14)		

### 3-Chloro-6-ethyl-5,7,8-trimethyl-2-nitro-4,4-diphenyl-4-bora-3a,4a-diaza-s-indacene (5d) Hydrogen-bond geometry (Å, º)

**Table:**

*D*—H···*A*	*D*—H	H···*A*	*D*···*A*	*D*—H···*A*
C19—H19*A*···O2^i^	0.95	2.43	3.365 (4)	168
C19—H19*A*···O2*A*^i^	0.95	2.36	3.238 (13)	154

Symmetry code: (i) *x*−1/2, −*y*+3/2, *z*−1/2.
